# Tyrosine O-sulfation proteoforms affect HIV-1 monoclonal antibody potency

**DOI:** 10.1038/s41598-022-12423-x

**Published:** 2022-05-19

**Authors:** Cindy X. Cai, Nicole A. Doria-Rose, Nicole A. Schneck, Vera B. Ivleva, Brad Tippett, William R. Shadrick, Sarah O’Connell, Jonathan W. Cooper, Zachary Schneiderman, Baoshan Zhang, Daniel B. Gowetski, Daniel Blackstock, Jacob Demirji, Bob C. Lin, Jason Gorman, Tracy Liu, Yile Li, Adrian B. McDermott, Peter D. Kwong, Kevin Carlton, Jason G. Gall, Q. Paula Lei

**Affiliations:** Vaccine Research Center, National Institute of Allergy and Infectious Diseases, National Institutes of Health, 9 West Watkins Mill Rd, Gaithersburg, MD 20878 USA

**Keywords:** Biochemistry, Biological techniques, Biotechnology, Structural biology

## Abstract

CAP256V2LS, a broadly neutralizing monoclonal antibody (bNAb), is being pursued as a promising drug for HIV-1 prevention. The total level of tyrosine-*O*-sulfation, a post-translational modification, was known to play a key role for antibody biological activity. More importantly, here wedescribe for the first time the significance of the tyrosine-*O*-sulfation proteoforms. We developed a hydrophobic interaction chromatography (HIC) method to separate and quantify different sulfation proteoforms, which led to the direct functionality assessment of tyrosine-sulfated species. The fully sulfated (4-SO_3_) proteoform demonstrated the highest in vitro relative antigen binding potency and neutralization efficiency against a panel of HIV-1 viruses. Interestingly, highly variable levels of 4-SO_3_ were produced by different clonal CHO cell lines, which helped the bNAb process development towards production of a highly potent CAP256V2LS clinical product with high 4-SO_3_ proteoform. This study presents powerful insight for any biotherapeutic protein development where sulfation may play an important role in product efficacy.

## Introduction

Recent discoveries of broadly neutralizing monoclonal antibodies (bNAbs) known for their high specificity of recognition on the HIV-1 viral envelope glycoprotein (Env) have provided new hope to suppress and to prevent active HIV-1 infection. Promising results have been shown not only in in vitro studies and animal models, but also in recent human clinical trials^1–4^. bNAbs offer the potential for next-generation treatments for HIV-1 patients to eliminate reservoir cells with lower toxicity and improved pharmacokinetics^4–8^. For any monoclonal antibodies, factors such as post-translational modifications (PTMs) are critical for protein biological functionality, thus an in-depth understanding and controlling of protein PTMs is very important for developing drugs against HIV-1^9–11^. As a result, many engineering designs have been applied to generate targeted PTMs for improving the antigen-binding surface of bNAb^12–14^. Multiple reports indicate that tyrosine-*O*-sulfation, a negative charged sulfo group (SO_3_^-^, labeled as SO_3_ in the following), of bNAbs may help establish a more robust intermolecular interaction between bNAbs and the antigen-binding site V1V2 domain of Env^15–18^.

CAP256V2LS, a bNAb targeting HIV-1 V1V2 at the Env trimer apex, was developed for clinical antibody studies. Its parent, CAP256-VRC26.25, is broadly cross reactive, particularly against non-clade B isolates, which had proven to be exceptionally potent and with good coverage against clade C viruses, the dominant HIV-1 clade in sub-Saharan Africa^19–22^. The CAP256V2LS construct was reengineered from its original wild type CAP256-VRC26.25 by an addition of the LS mutation in the Fc portion of the heavy chain to increase half-life in vivo and by an amino acid mutation from K117 to A to resist protease cleavage in cell culture media^21,23^ [Doria-Rose, submitted to Scientific Reports]. Both the parental molecule^19^ and the K to A mutant [Doria-Rose, submitted to Scientific Reports] neutralized 59% of a 208-strain multiclade panel at an IC_50_ < 50 µg/ml and 41% at a stringent cutoff of IC_80_ < 1 µg/ml. These values are higher for clade C, with 70% coverage at IC_50_ < 50 µg/ml, median IC_50_ = 0.004 µg/ml, and IC_80_ = 0.01 µg/ml.

Cryo-electron microscopy (cryo-EM) and X-ray crystallographic studies of its parental antibody CAP256-VRC26.25 confirm the presence of two potential sulfated tyrosine amino acid residues, 100 h and 100i, in the CDRH3 region of each antigen-binding fragment (Fab)^17,19^. Thus, up to four sulfated tyrosine residues in each full IgG molecule at the tip of CDR H3 (Figure S1) are associated with critical antigen-binding interactions.

The structure–function correlation of sulfation level and their corresponding binding and neutralization efficacy have previously been explored, which proved that higher level of sulfation correlated to higher antigen binding activity. Some studies have been concentrated on analyzing the global sulfation levels in a mixture of multiple sulfated proteoforms. Other studies have used the mutation of tyrosines to phenylalanines or inhibition of endogenous tyrosylprotein sulfotransferase (TPSTs) to eliminate the sulfation moieties for comparison of sulfated and un-sulfated species^24^. However, bioactivities directly correlated to individual sulfated species have not been studied due to the challenge of separating antibody proteoforms for a 150-kDa protein with only slight structural differences. Here we developed a hydrophobic interaction chromatography (HIC) method to fractionate different tyrosine-sulfated proteoforms (including the non-sulfated proteoform, for a total of 5 proteoforms). This new HIC method allowed separation of tyrosine-sulfated species, provided the potential to study the corresponding antigen binding efficacies of each proteoform, and was applied towards improving the activity of CAP56V2LS via assessing the level of sulfation modifications during CHO cell line and process development. We successfully characterized and produced a clinical antibody product with maximal sulfation. This advancement in characterization offers a valuable tool to the biopharmaceutical field for production of a mAb whenever sulfation is important in bioactivity.

## Materials and methods

The following reagents were LC–MS grade: water (Omnisolv, Billerica, MA), acetonitrile (ACN, J. T. Baker, Center Valley, PA), and formic acid (Thermo Fisher Scientific, Rockford, IL). All other chemicals were analytical grade: dithiothreitol (DTT, G Bioscience, St. Louis, MO), 0.5 M sodium phosphate buffer (Alfa Aesar, Haverhill, MA), and 5 M NaCl (Lonza, Alpharetta, GA). IdeS and PNGase F were from Promega (Madison, WI). Rapid PNGase F was from New England Lab (Woburn, MA).

### Cell culture method

A single vial of the CAP256V2LS clone, derived from CHO-DG44 cells, was thawed and used to inoculate 50 ml of ActiCHO P media (Cytiva, Piscataway, NJ) in a 250 ml shake flask to commence cell expansion. Cultures were expanded over the course of four passages, targeting a Viable Cell Density (VCD) between 3.75 and 4.50 × 10^6^ cells/ml per passage in shaking incubator, with each passage requiring 3 or 4 days. 3 l (Applikon, Scheidem, Netherlands) or 50 l SUB Bioreactors (ThermoFisher, Waltham MA) were inoculated at a target Viable Cell Density (VCD) of about 0.6 × 106 cells/ml using ActiCHO P basal media. The cultures were supplemented with 3% Feed-A and 0.3% Feed-B (Cytiva, Piscataway, NJ) starting on day 3. Glucose levels were sampled daily, and glucose was added targeting between 1.0 and 2.0 g/l. pH was set to 7.0 ± 0.3, dissolved oxygen was set to 50% and agitation was set to 120 rpm. The cell culture was harvested at day 14.

### Generation of load material from butyl HP chromatography

Cell culture harvest was clarified by a two-stage depth filtration train of Clarisolve 20MS and F0HC (MilliporeSigma, Burlington, MA) followed by sterile filtration using a Sartopore 0.8/0.2 μm filter (Sartorius Stedim, Goettingen, Germany), operated in tandem. All chromatography was performed on an AKTA AVANT 25 (GE Healthcare). Clarified harvest was loaded onto a Protein A column (TOYOPEARL AF-rProtein A HC-650F, Tosoh Bioscience, King of Prussia, PA) and eluted with 100 mM glycine–HCl, pH 3.5. The material was then conditioned to 185 mM sodium chloride using 5 M NaCl stock solution (Lonza, Walkersville, MD) and titrated to pH 9.0 using 2 M glycine-OH, pH 10.5. The conditioned material was then purified via TOYOPEARL NH2-750F anion-exchange chromatography (Tosoh Biosciences), operated in flow-through mode using 50 mM Tris–HCl pH 8.0, 200 mM NaCl as the column equilibration and load chase buffer.

### Preparative butyl HP sepharose chromatography

The anion exchange product was conditioned to 20 mM Tris–HCl pH 8.0, 3.0 M NaCl by addition of 5 M NaCl stock solution. A 4.7 ml column volume (CV) Butyl Sepharose HP, 10 cm bed height, HiScreen column (Cytiva, Marlborough, MA) was equilibrated with 50 mm Tris–HCl pH 8.0, 3 M NaCl, followed by loading the conditioned anion-exchange product to a target resin capacity of 5 mg/ml. Column wash and elution utilized the system gradient feature, using 50 mm Tris–HCl pH 8.0, 3 M NaCl (Buffer A) and 50 mm Tris–HCl pH 8.0 (Buffer B). A 5 CV wash was performed at 47% B (~ 1380 mM NaCl) and elution was executed across a 50–100% B 25 CV gradient. The elution peaks were collected in 2 ml fractions and stored at 2–8 °C prior to analytical testing.

### Analytical HIC-UV method for quantification

The Acquity H-class Bio UPLC system (Waters, Milford, MA) was operated using Empower 3.0. Mobile phases A (10 mM sodium phosphate buffer with 4 M NaCl, pH 7.0) and B (10 mM sodium phosphate buffer, pH 7.0) in water was delivered at the flow rate of 0.5 ml/min. Samples were injected onto a ProPac HIC-10 column (Thermo Fisher Scientific, 5 µm, 300 A, 4.6 × 100 mm) at ambient temperature with the detection UV wavelength at 280 nm. The system was equilibrated at 10% B for 2.0 min. MPB was increased linearly to 100% (2.0–20.0 min) and held at 20.0–21.0 min. The column was then equilibrated with 10% B in 22.1–27.0 min range. The total run time is 27.0 min. The detected UV peak area was applied for the relative ratio quantification of sulfation levels.

### RPLC-MS for peak identification

The UPLC system operated by MassLynx v.4.1 was coupled with a Q Exactive HF mass spectrometer (Thermo-Fisher Scientific) that was operated using XCalibur v.4.1. Mobile phases A and B consisted of 0.1% (v/v) formic in water and ACN, respectively, delivering at the flow rate of 0.2 ml/min. Positive ionization mode was applied with a mass detection range of 550–3000 Da at the resolution 15,000 FWHM. The capillary voltage was at 3.5 kV, capillary temperature was set to 350 °C, and the probe heater temperature was at 400 °C. BioPharma Finder 2.0 was applied for mass deconvolution. For intact mass analysis, two-step deglycosylation protocol was applied to completely remove the glycans^25^. 20 µg of each CAP256V2LS sample was incubated with 1 μL of PNGase F at 37 °C (pH 7.8) for 2 h followed by the digestion using 1 μl of Rapid PNGase F (5 × diluted) at 55 °C (pH 7.8) for 15 min. Samples were injected onto a BEH C4 column (Acquity, 1.7 µm, 2.1 mm × 50 mm) heated at 80 °C. The UPLC system was equilibrated at 25% B for 2.0 min. MPB was increased linearly to 33.5% at 7.0 min and to 90% at 7.0–9.0 min. The column was then equilibrated with 25% B in 9.1–12.0 min range. The total run time is 12.0 min.

### Size exclusion chromatography (SEC) method

The UPLC system operated at an isocratic flow rate of 0.4 ml/min with 2 × PBS mobile phase using ACQUITY UPLC BEH 200 Å SEC column (1.7 μm, 4.6 × 150 mm; Waters, Milford, MA). Running time of each sample was 12 min with the detection wavelength at 280 nm.

### HIV-1 antibody–antigen binding assay

CAP256V2LS antibody–antigen-binding assays were conducted in duplicate using 90 μL per well in a 384-well tilted-bottom microtiter plate. Each testing well contained CAP256V2LS with concentrations ranging from 0 to 10 μg/ml in assay buffer (20 mM Sodium Phosphate, 75 mM Arginine, 100 mM NaCl, 3% Sucrose, 0.01% PS80, pH 7). CAP256V2LS was bound to a Protein A biosensor which was then washed in assay buffer to remove un-bound antibody. The sensor-antibody complex was then dipped in a well containing HIV-1 antigen (BG505 SOSIP.664) at 100 µg/ml. The binding results for the CAP256V2LS- HIV-1 antigen interaction was measured on an Octet RED384 (Forte Bio) and given as nanometer shift. Increases in bound CAP256V2LS- HIV-1 antigen complexes, results in greater wavelength shifts detected using Octet analysis. As the results, the antibody-antigen binding curve was obtained by plotting the wavelength shifts versus the antibody concentration. The resulting data were log-transformed, and a dose–response curve was fitted using the four parameters symmetrical equation “log(agonist) vs. response-Variable slope (four parameters)” with default constraints in GraphPad analysis software Prism Version 9.0. Relative potency (%RP) was determined using the built-in equation “EC50 shift, X is log(concentration)”, using default constraints. Sample potencies were determined by the following equation: relative antigen binding potency = 100 × (1/EC_50_ ratio). SE (standard error) is reported by Prism software defining the EC_50_ reporting value range generated by the full curve analysis.

### HIV-1 ENV-pseudovirus neutralization assay

The HIV-1 ENV-pseudovirus neutralization assays were performed on integrated automation platforms consists of Biomek FX liquid handler from Beckman Coulter, ambient temperature labware hotel (Thermo Scientific), 37 °C incubator (Thermo Scientific/Liconic), and Molecular Devices Paradigm Multimode reader as described^26^. The automated assay methods are operated through Beckman Coulter SAMI EX software. On day 1, samples are diluted starting at 50 µg/ml and 9 × 5-fold serial dilutions in D10 culture medium (10% FBS, DMEM, gentamycin) in 384-well deep-well plate and stamped into 4 identical tissue culture plates at 30 µl per well (Thermo Scientific Nunc 384-well polystyrene plates, Cell culture surface; w/lid black Part No. 164564). Env-pseudotyped viruses with the HIV SG3dEnv backbone^26^ were diluted in D10 and added at 30 µl per well into tissue culture plate containing the serially diluted sample, followed by 45 min incubation at 37 °C w/ 5% CO_2_. TZM-bl reporter cells were added at 3000 cell per well in 20 µl into virus/sample tissue culture plate, followed by 48–54-h incubation at 37 °C w/ 5% CO_2_. On day 3 (48 h from Day 1), 50 µl of culture medium was removed from plate and 30 µl of luciferase substrate (Perkin Elmer Britelite Plus Cat. No. 6066769) was added. The substrate and cell culture were incubated at room temperature for 2 min and luminescence signal (RLU, relative luminescence unit) was measured in Paradigm Multimode reader. The neutralization percentage of test sample was determined by normalizing the test sample RLU to the RLU of virus and cell control well with the following calculation: percent neutralization = [(Test wells − Average Cell Control Wells) − (Average Virus Control Wells − Average Cell Control Wells)] ÷ (Average Virus Control Wells − Average Cell Control Wells) × 100. The neutralization curve fit was generated on NAB analysis module on Labkey web-based server with 5 parameter non-linear regression. Neutralizing antibody titers are expressed as the reciprocal of the serum dilution required to reduce RLU by a designated percentage and reported as inhibition concentration (IC) or inhibition dosage (ID) values.

## Results

**Figure 1:**
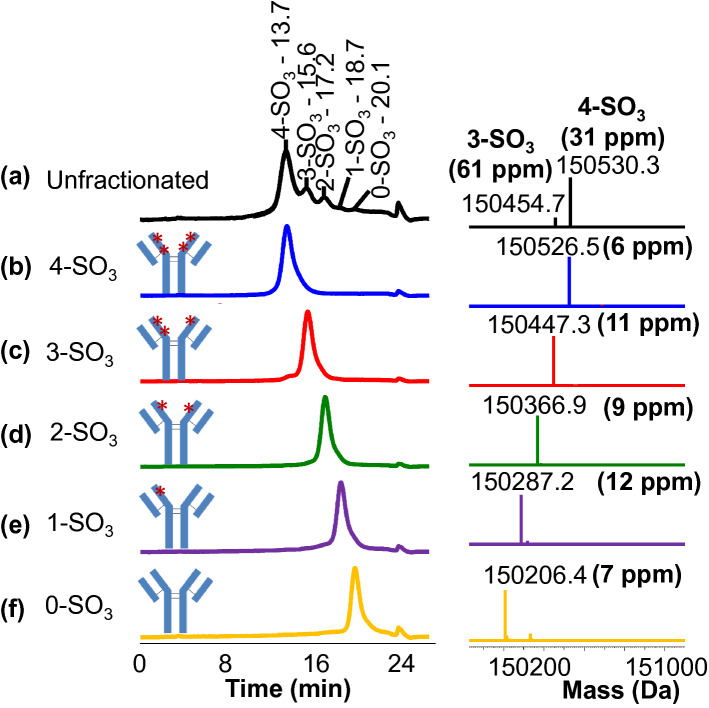
HIC-UV chromatograms and RPLC-mass spectrometry analysis distinguishing CAP256V2LS sulfation proteoforms. Traces on the left are CAP256V2LS analyzed by HIC-UV. (y-axis is the UV absorbance, normalized to the highest peak intensity in each chromatogram). On the right, deconvoluted Reversed phase coupled with intact mass analysis from each peak fraction corresponding to the left elution after deglycosylation. (y-axis is the mass intensity, normalized to the highest peak in each mass spectrum.) (**a**) Unfractionated CAP256V2LS showed five peaks on HIC-UV chromatograms, with the peak of 4-SO_3_ and 3-SO_3_ species detected in RPLC-MS. (**b**) 4-SO_3_ eluted first at 13.7 min with mass confirmed at 150,526.5 Da. (**c**) 3-SO_3_ eluted at 15.6 min with mass of 150,447.3 Da. (**d**) 2-SO_3_ eluted at 17.2 min with mass observed at 150,366.9 Da. (**e**) 1-SO_3_ eluted at 18.7 min with mass detected at 150,287.2 Da. (**f**) 0-SO_3_ eluted last at 20.1 min with mass measured at 150,206.4 Da.

Separation and identification of different sulfated proteoforms was completed using HIC-UV and Reversed-phased Liquid Chromatography coupled with Mass spectrometry detection (RPLC-MS). The addition of the sulfo group to tyrosine decreases protein hydrophobicity^27,28^ incrementally with more sulfation modifications, leading to different elution times from HIC for differently sulfated variants. Based on the structural data, five proteoforms of CAP256V2LS are possible: zero (0-SO_3_), one (1-SO_3_), two (2-SO_3_), three (3-SO_3_), and four (4-SO_3_) tyrosine sulfations, and the all forms of CAP56V2LS were separated using the newly developed HIC-UV method (Fig. 1a). The zero (0-SO_3_) and one (1-SO_3_) species were observed at very low intensities < 10%. The analytical method was then transferred to a preparative HIC column in which the five peaks (Figure S2) could be clearly observed and fractionated for additional analyses. Purity of each fraction was verified to be > 89% by re-injecting the fractions to the analytical HIC-UV analysis (Fig. 1b–f).

RPLC-MS was applied for peak identifications and confirmation. Unfractionated sample and each individual fraction were deglycosylated to minimize peak heterogeneity prior to the RPLC-MS analysis (Fig. 1). The deconvoluted intact masses for the five fractions were observed at 150,526.5 Da, 150,447.3 Da, 150,366.9 Da, 150,287.2 Da and 150,206.4 Da, respectively. Specifically, the 80 Da mass shifts reveal that CAP256V2LS had been modified with four, three, two, one, and zero sulfo group(s). Mass errors were > 30 ppm for 3-SO_3_ and 4-SO_3_ species due to the high level of heterogeneity of unfractionated CAP256V2LS. However, the mass accuracy was improved to < 12 ppm when each fraction was analyzed, likely due to less interference with a more pure sample in MS ionizations compared to the unfractionated sample.

### Bioactivity increases with higher tyrosine sulfation occupancy

**Figure 2:**
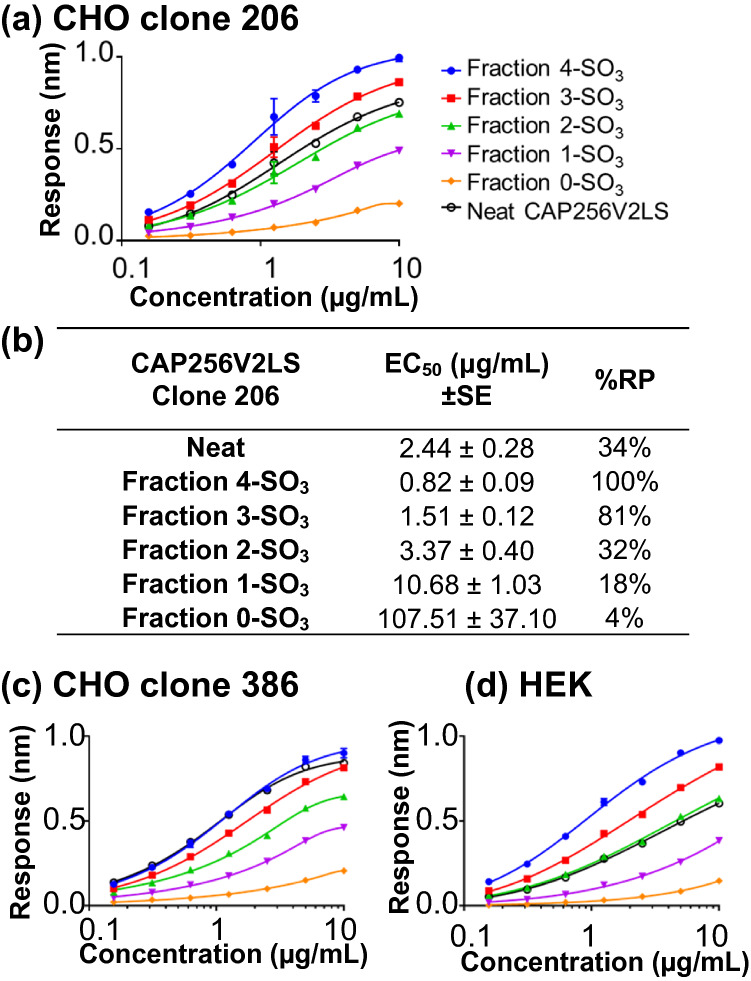
Increased levels of CAP256V2LS tyrosine sulfation correspond to increased antigen binding. (**a**) Octet antigen-binding curve for fractions of CAP256V2LS generated from CHO clone 206. (**b**) Relative antigen binding potency (%RP) for each level of tyrosine sulfation proteoform of CAP256V2LS. Octet antigen-binding curve for fractions of (**c**) CHO clone 386 and (**d**) CAP256V2LS expressed in HEK-293 cells. %RPs were normalized against the highest 4-SO_3_ proteoform from the same parent material. Binding curve data points shown as average with error bars are from two independently prepared experiments.

To determine the impact of sulfation level on biological activity, we assessed binding of sulfation proteoforms of CAP256V2LS to an HIV antigen. Proteoforms were fractionated from CAP256V2LS produced in several cell lines: HEK293-expi, and several clones of stable transfected Chinese hamster ovary (CHO) cells. The latter were produced as part of a program for large-scale antibody production. One CHO clone 206 was first purified using a protein A column followed by the HIC fractionation method. Binding of each fraction to the native-like trimeric HIV-1 Env protein BG505 SOSIP.664 was measured by biolayer interferometry using Octet^19^. Lower EC_50_ (in μg/ml) indicates stronger relative potency (RP%) for a mAb via binding assessment (Fig. 2). There was an increasing trend in binding efficiency EC_50_, ranging from 107.51 to 0.82 μg/ml for 0-SO_3_ and 4-SO_3_, respectively (Fig. 2a,b). After normalization against 4-SO_3_, each sulfation level clearly showed decreased %RP with decreased sulfation occupancy; 81% for 3-SO_3_, 32% for 2-SO_3_, 18% for 1-SO_3_, and down to almost zero (4%) when the CAP256V2LS was completely free of sulfation (at 0-SO_3_). We infer this is due to more binding sites available in the 4-SO_3_ form than in other sulfated form. The observed EC_50_ of binding for the 4-SO_3_ proteoform was about 131-fold lower compared to the 0-SO_3_ species, with intermediate forms trending appropriately. A similar trend in antigen binding was observed when comparing material purified either from CHO clone 386 (Fig. 2c, Table S1) or transiently transfected HEK293-expi cells (Fig. 2d, Table S2). Consistently among all three sources, the unfractionated material of CAP256V2LS (a mixture of multiple proteoforms) showed an intermediate weighted average binding potency, consistent with having contributions from the different proteoforms within each sample.

**Figure 3:**
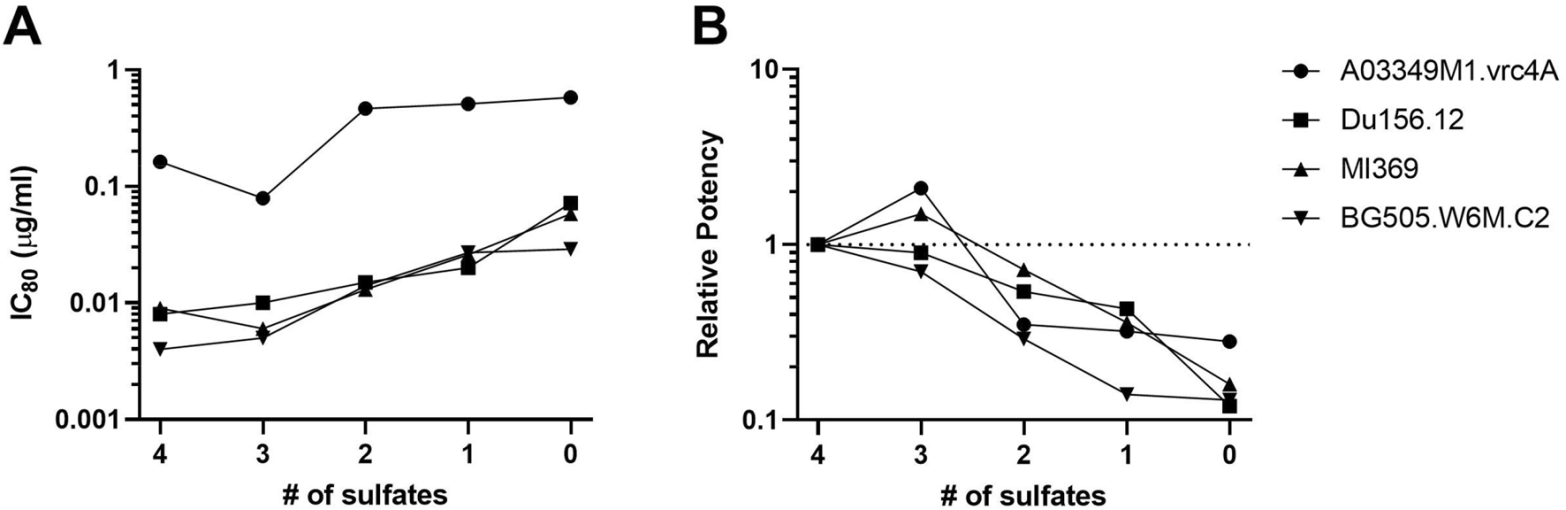
Neutralization potency of CAP256V2LS versus tyrosine sulfation occupancy. Sulfation fractions from CHO clone 206 were assayed in one experiment for neutralization activity against 4 Env-pseudoviruses, (**A**) IC_80_ and (**B**) relative neutralization potency of each virus, calculated as the IC_80_ of the 4-SO_3_ proteoform divided by the IC_80_ of the tested fraction.

To test the impact of sulfation on neutralization, we assayed the fractionated material in an HIV-1 Env-pseudovirus neutralization assay. Pseudoviruses containing diverse Envs were selected to represent a range of neutralization resistance: A03349M1_VRC4A (Clade D), Du156.12 (Clade C), MI369 and BG505_W6M_C2 (Clade A), as well as SVA.MLV (non-HIV control, Murine Leukemia Virus). This panel was first used to test the neutralizing activity of CAP256V2LS sulfated proteoforms fractioned from clone 206. In the neutralization assay, a lower IC_80_ value (µg/ml) indicates stronger neutralization. For each pseudovirus, the lowest activity was noted for the 0-SO_3_ proteoform, with a loss of potency as much as eightfold compared to the 4-SO_3_ proteoform (Fig. 3; Tables S1, S2). The number of sulfates correlated with potency against Du156 and BG505.W6M.c2 (p = 0.017, Spearman’s rho, for each) and trended to significance for A03349M1_VRC4A and MI369. Figure 3 summarizes the neutralization results of five sulfated proteoforms (including the unfractionated material without separation) against the four virus subtypes; representative curves are shown in Figure S3. This neutralization study was performed again on sulfated proteoform fractions from CHO clone 386 and on HEK-293 material, where the trend was repeated (Tables S1, S2). Based on both relative binding potency and neutralization assessment, we conclude that higher sulfation leads to higher activity for bNAb CAP256V2LS.

### Stability analysis for 4-SO_3_

Some reports show that sulfotyrosine readily degrades under a forced degradation condition of temperature 25–70 °C and/or low pH 1–3^29,30^. To ensure that product quality is not altered during process purification, material transfer, and storage, stability of tyrosine sulfation in CAP256V2LS was evaluated under two stressed conditions: temperature excursion and low pH. To mimic temperature fluctuation during sample storage and handling processes, CAP256V2LS was stored at 25 °C and 40 °C for up to 2 weeks. Stressed samples along with a CAP256V2LS reference standard (starting material stored at − 80 °C) were analyzed using the HIC-UV method. The abundance of 4-SO_3_ was not affected by the heat-stress conditions (Table S3). This indicated that degradation of the tyrosine sulfation level is unlikely to occur at temperatures between 25 and 40 °C within 2 weeks. Secondly, the effect of low pH on tyrosine sulfation stability was evaluated for CAP256V2LS, mimicking the low pH conditions during purification by Protein A. The tyrosine sulfation profile remained comparable after 2 h-incubation at pH 3.3 (Figure S4). This showed that a protein A elution step is unlikely to cause changes in sulfation levels for CAP256V2LS. Overall, we concluded that process purification steps would not adversely affect sulfation levels for CAP256V2LS.

### Clone screening and selection for high fully sulfation proteoform

**Figure 4:**
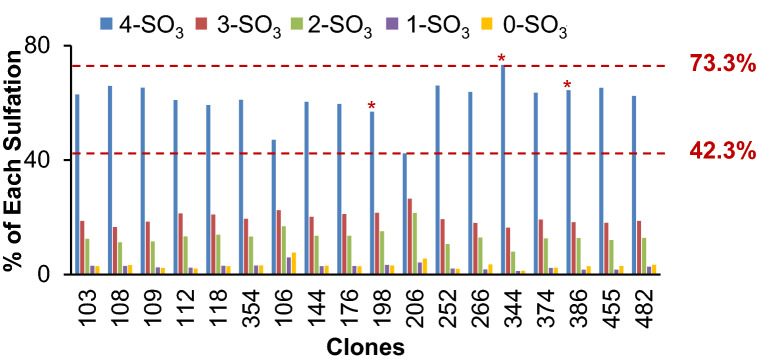
Abundance of CAP256V2LS sulfation proteoforms varied with different CHO cell clones. Sulfation proteoform quantitation of CAP256V2LS expressed from 18 CHO clonal cell lines cultured at 250-ml scale was performed once for each sample. Asterisk marks the three top clones selected for subsequent 3-l scale studies.

Given the differences in CAP256V2LS sulfation levels observed between CHO clones 206 and 386 (Fig. 4), several more CHO clones were screened for CAP256V2LS sulfation. Eighteen clones were expanded to 250 ml culture and analyzed by the HIC-UV method. Overall, 4-SO_3_ was the dominant proteoform in all clones, ranging from 42.3% to 73.3% (Fig. 4). By evaluating all factors together including high CAP256V2LS expression, low aggregation, low fragmentation levels (data not shown), and high 4-SO_3_ percentage (Fig. 4), three top clones (344, 198 and 386) were selected for additional cell culture development and process scale-up. Four replicate 3-l bioreactor cultures were performed for each of the three clones and CAP256V2LS was assessed for aggregation and sulfation percentage individually (Table 1). Four replicates from each clone were pool into one lot to test for relative binding potency and neutralization potency for four psuedoviruses (Table 1). Among the three clones at scale-up development, aggregation was tested at 5.7%, 4.9% and 2.6% for clone 198, 344 and 386, which while slightly different, was unlikely to cause the significant difference for bioactivities. The sulfation profile of CAP256V2LS expressed by two of the clones had substantially different 4-SO3 percentages at the different cell culture scales. CAP256V2LS expressed by clone 386 had the highest 4-SO_3_ percentage at the 3-l scale, 87.0% (Table 1), compared to the 250 ml-scale, 64.6% (Fig. 4). In contrast, clone 344 had the highest 4-SO_3_ percentage of all 18 clones at the 250-ml scale, 73.3% (Fig. 4), but only 58.1% (Table 1) at the 3-l scale. The sulfation profile of the clone 198 remained similar. Among the three clones evaluated at 3-l scale, CAP256V2LS expressed by clone 386 had the highest 4-SO_3_ level and concurrently showed the highest antigen-binding and neutralization efficiencies (Table 1). Clone 386 was selected for further process development.

**Table 1:** Aggregation, %4-SO_3_ levels and the corresponding in vitro potency for the top three clones.

CAP256V2LS clone	Aggregation % (n = 4)	4-SO_3_% (n = 4)	%RP	Neutralization efficiency IC_80_			
				A03349M1_VRC4A	Du156.12	MI369	SVA.MLV
198	5.7 ± 0.5	54.7 ± 5.3	70	0.093	0.019	0.014	> 50
344	4.9 ± 0.8	58.1 ± 1.8	84	0.059	0.012	0.010	> 50
386	2.6 ± 0.1	87.0 ± 0.3	99	0.025	0.009	0.007	> 50

(%RP, a single value from the curve, is normalized against an interim CAP256V2LS reference standard. Neutralization efficiency IC_80_ is a single value derived from interpolation of 80% signal reduction on a 10 point serially diluted point curve fit via 5-PL non-regression analysis).

### Process consistency and improvement evaluation

**Figure 5:**
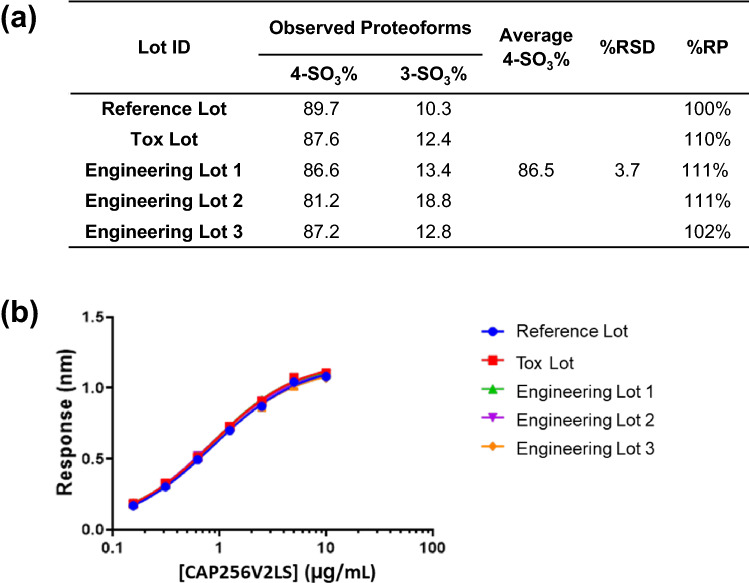
Lot-to-lot consistency of CAP256V2LS sulfation profile. Five independently produced lots of CAP256V2LS at the 50-l scale included one reference lot, one toxicology lot and three consistency-run lots. (**a**) % Relative potency values are shown for binding to HIV-1 antigen accordingly to procedures detailed in materials and methods. Reported value %RP for all lots is compared to reference lot. (**b**) Octet antigen-binding curves.

Five lots of CAP256V2LS at 50-l bioreactor scale were generated from clone 386, one for the Reference Standard, one for a toxicology study and the three others as consistency runs. The sulfation distributions were evaluated for all five lots for product consistency. 4-SO_3_ and 3-SO_3_ were the only species detected in all five lots, with relative % 4-SO_3_ proteoform being above 81% (Fig. 5a). The relative binding potencies among the five lots were comparable, ranging from 100 to 111% (Fig. 5b). The neutralization strength against four psuedoviruses (IC_80_) were also comparable among the 5 lots, within assay variability (Table S4). Lastly, clinical trial material was produced following cGMP at a 1000-l production scale. The clinical trial material had a 4-SO_3_ level of 88.7% and a 3-SO_3_ level of 11.3%, confirming manufacturing consistency with process scale-up. Compared to the HEK-293 product material with relatively lower 4-SO_3_ level at 41.4% (Figure S5), clinical-grade material produced from the selected high yield CHO clone 386 CAP256V2LS (with 4-SO_3_ level of > 81%) showed improved neutralization potency, with a fourfold lower median IC_50_ value [Doria-Rose, submitted to Scientific Reports].

## Discussion

Prior to this work, some semi-quantitative analyses on sulfation abundance were obtained using subunit or peptide mapping mass spectrometry analyses, in which the un-sulfated and sulfated Fab’s were differentiated and the relative abundance via peak heights was calculated^31–33^. However, using mass spectrometry as a quantitative approach has its limitations. Ionization efficiency varies among different species, leading to a potentially biased quantification. In this work, we used the HIC-UV method to isolate and detect the different CAP256V2LS proteoforms with four, three, two, one and zero sulfo group(s). The separation of the sulfated proteoforms provided the foundation for direct understanding of the structure–function relationship, which was not reported previously. A stoichiometry of 1:1 for Fab:HIV-1 trimer binding was reported in literatures for CAP256V2LS binding to antigen^17^. That being said two Fabs from one bNAb together can potentially bind to two trimers at the same time, which leads to a hypothesis that total antigen-binding activity of CAP256V2LS comes from the sum of two Fabs. If each heavy chain containing two sulfation modifications contributes equally to CAP256V2LS binding activity for 4-SO_3_ proteoform, the RP% is 50% for each arm and 100% for both heavy chains together. In the case of the 3-SO_3_ species, one Fab region of CAP256V2LS keeps the maximum binding activity (50%). However, the other one loses a partial negative charge, which decreases one-arm interaction with the antigen more than half (reduced from 50 to < 25%). It reduces the CAP256V2LS-BG505 SOSIP.664 binding complex and leads the RP% fall in the range 50–75%, which are close to our detected values (52–81%). 2-SO_3_ proteoform bears one sulfation on each Fab (Figure S6), presenting significant antigen binding reduction for each arm (< 25%) and sum of both Fabs (< 50%), which matches our measurements (23–32%). With further reduction of sulfation levels, 1-SO_3_ continues the trend of an extensive decline of antigen-binding activity to < 25%, (the binding activity < 25% and 0% for the heavy chain with one and 0 sulfation, respectively), which is consistent with the observed values between 8 and 18%. When neither heavy chain contains *O*-sulfation modification, the binding activity is expected to be 0%, which aligns with the measurements (1–4%). Based on these results, we propose that existence of both sulfated tyrosine moieties on two heavy chains are critical for CAP256V2LS binding to HIV-1 Env. This sulfation occupancy study supports the previous findings in regard to the critical role of the tyrosine sulfation modification, i.e., the heavy chain bearing sufficient negative sulfo groups is the key to maintain electrostatic contact with the binding pocket on the HIV-1 trimer apex. More importantly, our results clearly present molecular binding efficacy significantly dropped with the loss of only one sulfo group and further decreased with reduced sulfation occupancy. Without sulfation, the measured binding of CAP256V2LS to its antigen by an Octet method goes down to almost zero. Our work provides the capability to examine in more detail the influence of sulfation on bNAb activity, from antigen-binding to neutralization efficiency. As a result, the sulfation occupancy is more important than total sulfation amount for ensuring high antigen binding effectiveness, which is critical for product development. This discovery supports our assertion that sulfation level can be a product potential critical quality attribute (pCQA) and should be controlled and monitored during the clinical material manufacturing process^34–36^.

Multiple labs have demonstrated the importance of sulfation modification on the function of HIV-1 mAbs, such as PGT145 and PGMD1400, and worked to enhance the sulfation modification for greater activity^37,38^. Over-expression of TPST to increase sulfation level was observed by using tryptic digestion followed by peptide mapping analysis^39,40^, however, low amount of double sulfated peptide (< 25%) indicated the low abundant 4-SO_3_ species^32^. As a result, the enzymatic approach has its limitation on increasing the sulfation level. Furthermore, enzyme catalysis using TPSTs was not reported and may not be feasible for commercial manufacturing (> 1 kg) due to unknown safety concerns and additional high costs. CHO cell lines have demonstrated suitability to provide sufficient bNAb material for its long-term clinical purpose^41–43^. For CHO clone selection during the process development, previously published approaches such as mass spectrometry analysis, the binding (via anti-sulfotyrosine antibody) or radiolabeling assays has very limited application since they can only provide analysis on the global sulfation level^33,44–46^. Whereas our newly developed HIC method can not only provide accurate sulfation profiling, but also allow the purification of individual proteomer for characterization, enabling the selection of a product with the highest possible HIV-1 neutralization potency.

The structure–function characterization data in this study strongly supports the necessity of monitoring the sulfation proteoforms of antibodies with potential sites for tyrosine sulfation. It has been widely reported that sulfotyrosine on a bNAb’s CDR H3 is common and advantageous for recognition of the positively charged V1V2 region on HIV-1 Env^47–50^. Structural studies have defined the mechanism of this dependence, with visualization of the key contacts with Env that are made by sulfated tyrosine residues on the bNAb^17,18^. Changes in 4-SO3 proteoform abundance were detected during process development scale-up, allowing for selection of a CHO clonal cell line that produced the highest level of sulfation under bioreactor culture conditions. Additionally, the HIC-UV method verified the stability of the sulfation profile through temperature fluctuation (mimicking storage and clinical handling excursion) and low pH stress (mimicking purification processes). Transfer of the manufacturing process to the clinical manufacturing site could have resulted in changes to the sulfation profile, and therefore potency, due to the larger scale of cell culture. However, the sulfation profile was consistent through manufacturing of the clinical material. Monitoring and controlling the 4-SO_3_ level will allow for a more consistent product, which is essential for successful product development.

## Conclusions

In summary, in-depth characterize of the sulfation proteoform has allowed us to draw a structural–functional correlation between sulfation and bioactivities of a potent bNAb. Through CHO-clone generation and selection, process development and product characterization, we have designed a process that preserves high levels of 4-SO_3_ in our clinical drug product. This clinical material is undergoing safety and pharmacokinetics evaluation in first-in-human studies in South Africa, which could be used in a pre- exposure prophylaxis strategy given its exceptional potency and breadth against the dominant HIV-1 clade C strains in sub-Saharan Africa^22^. Given these new perspectives on tyrosine sulfation that our study provides, we view percent level of 4-SO_3_ (full sulfation occupancy) as a potential critical quality attribute (pCQA) for CAP256V2LS and recommend evaluation of tyrosine sulfation occupancy on other antibodies where the sulfation might play a role in bioactivity.

## Supplementary Information

Supplementary Information.
