# Supplementary material for: Tyrosine O-sulfation proteoforms affect HIV-1 monoclonal antibody potency
Source: Sci Rep. 2022 May 19;12:8433. doi: 10.1038/s41598-022-12423-x (PMC9120178; doi:10.1038/s41598-022-12423-x)
Supplement: Supplementary file 1 — Supplementary Information. [file 41598_2022_12423_MOESM1_ESM.docx]

Supporting Information

**Tyrosine O-Sulfation Proteoforms Affect HIV-1 Monoclonal Antibody Potency**

Cindy X. Cai, Nicole A. Doria-Rose, Nicole A. Schneck, Vera B. Ivleva, Brad Tippett, William R. Shadrick, Sarah O’Connell, Jonathan W. Cooper, Zachary Schneiderman, Baoshan Zhang, Daniel B. Gowetski, Daniel Blackstock, Jacob Demirji, Bob C. Lin, Jason Gorman, Tracy Liu, Yile Li, Adrian B. McDermott, Peter D. Kwong, Kevin Carlton, Jason G. Gall, Q. Paula Lei*

Vaccine Research Center, National Institute of Allergy and Infectious Diseases, National Institutes of Health, Gaithersburg, MD, USA

*Q. Paula Lei, email: [paula.lei@nih.gov](mailto:paula.lei@nih.gov)

Address: 9 West Watkins Mill Rd, Gaithersburg, MD 20878 USA

Table of Contents

Figure S1. Structural characteristics of CAP256-VRC26.25.

Figure S2. Preparative scale HIC-UV chromatogram of CAP256V2LS with each fraction subsequently collected.

Table S1. Neutralization efficiency against a small panel of HIV-1 viruses and Octet binding potency for CAP256V2LS CHO clone 386.

**Table S2.** **Neutralization efficiency against small panel of HIV-1 viruses and Octet binding potency for CAP256V2LS CHO material.**

Figure S3. Neutralization plots from Labkey analysis of five different levels of sulfation for four panel of virus.

Figure S4. CAP256V2LS sulfation is not altered by low pH.

Table S3. HIC-UV Monitoring 4-SO_3_% levels for CAP256V2LS during elevated temperature stress.

Table S4. Neutralization efficiency against small panel of HIV viruses for 5 CAP256V2LS lots.

Figure S5. CAP256V2LS sulfation profile was detected for CAP256V2LS HEK-293 material using HIC-UV method.

Figure S6. Analysis of Fd sulfation profile for different sulfation proteoforms.


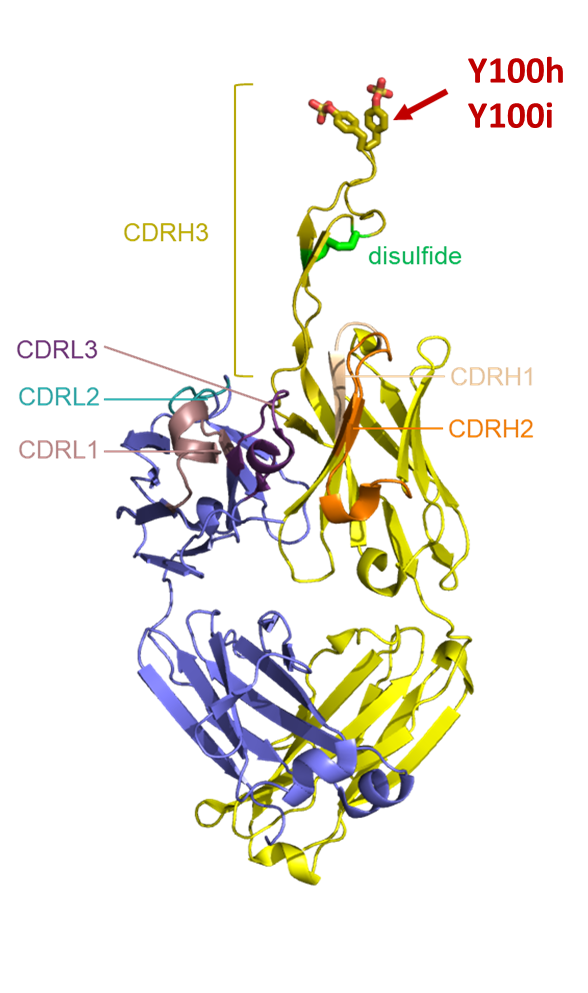


Figure S1 Structural characteristics of CAP256-VRC26.25. Crystal structure of the antigen-binding fragment (Fab) of CAP256-VRC26.25 shown in ribbon diagram representation. Tyrosine 100h and 100i at the CDR H3 region are labeled [17].


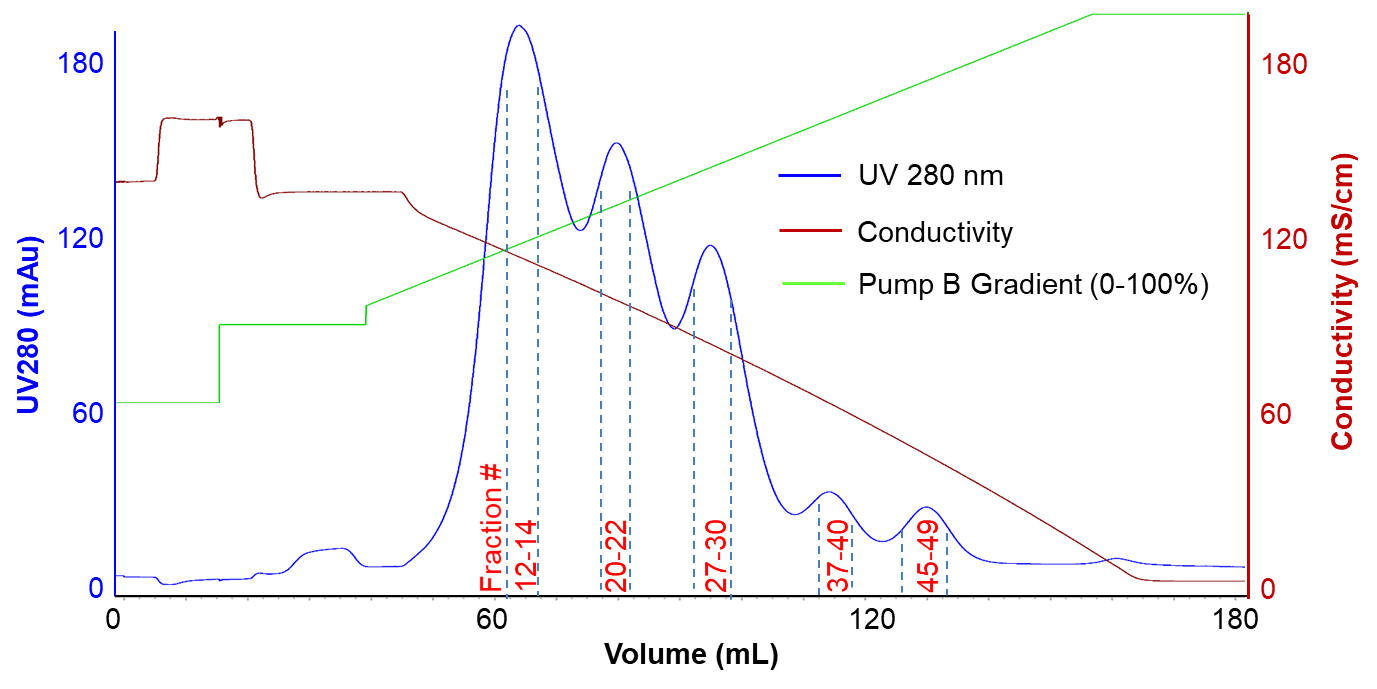


Figure S2 Preparative scale HIC-UV chromatogram of CAP256V2LS with each fraction subsequently collected. Pool 1 (fraction 12-14), pool 2 (fraction 20-22), pool 3 (fraction 27-30), pool 4 (fraction 37-40) and pool 5 (fraction 45-49) correlated to 4, 3, 2, 1, and 0-SO_3_ modifications, respectively.

Table S1 Neutralization efficiency against a small panel of HIV-1 viruses and Octet binding potency for CAP256V2LS CHO clone 386.

| **Samples** | **Neutralization efficiency IC80 (ug/mL)** | | | | **Relative binding potency** |
| --- | --- | --- | --- | --- | --- |
|  | **A03349M1_VRC4A** | **Du156.12** | **MI369** | **SVA.MLV** |  |
| **Neat** | 0.070 | 0.005 | 0.010 | >50 | 95% |
| **Fraction 4-SO_3_** | 0.042 | 0.004 | 0.005 | >50 | 100% |
| **Fraction 3-SO_3_** | 0.027 | 0.012 | 0.013 | >50 | 59% |
| **Fraction 2-SO_3_** | 0.485 | 0.028 | 0.024 | >50 | 29% |
| **Fraction 1-SO_3_** | 5.732 | 0.260 | 0.165 | >50 | 11% |
| **Fraction 0-SO**_3_ | 0.255 | 0.030 | 0.023 | >50 | 2% |

**Table S2** Neutralization efficiency against small panel of HIV-1 viruses and Octet binding potency for CAP256V2LS HEK-293 material.

| **Samples** | **Neutralization efficiency IC_80_ (ug/mL)** | | | | **Relative binding potency** |
| --- | --- | --- | --- | --- | --- |
|  | **A03349M1_VRC4A** | **Du156.12** | **MI369** | **SVA.MLV** |  |
| **Neat** | 0.064 | 0.007 | 0.007 | >50 | 39% |
| **Fraction 4-SO_3_** | 0.163 | 0.008 | 0.009 | >50 | 100% |
| **Fraction 3-SO_3_** | 0.079 | 0.010 | 0.006 | >50 | 52% |
| **Fraction 2-SO_3_** | 0.466 | 0.015 | 0.013 | >50 | 23% |
| **Fraction 1-SO_3_** | 0.511 | 0.020 | 0.026 | >50 | 8% |
| **Fraction 0-SO**_3_ | 0.580 | 0.072 | 0.058 | >50 | 1% |


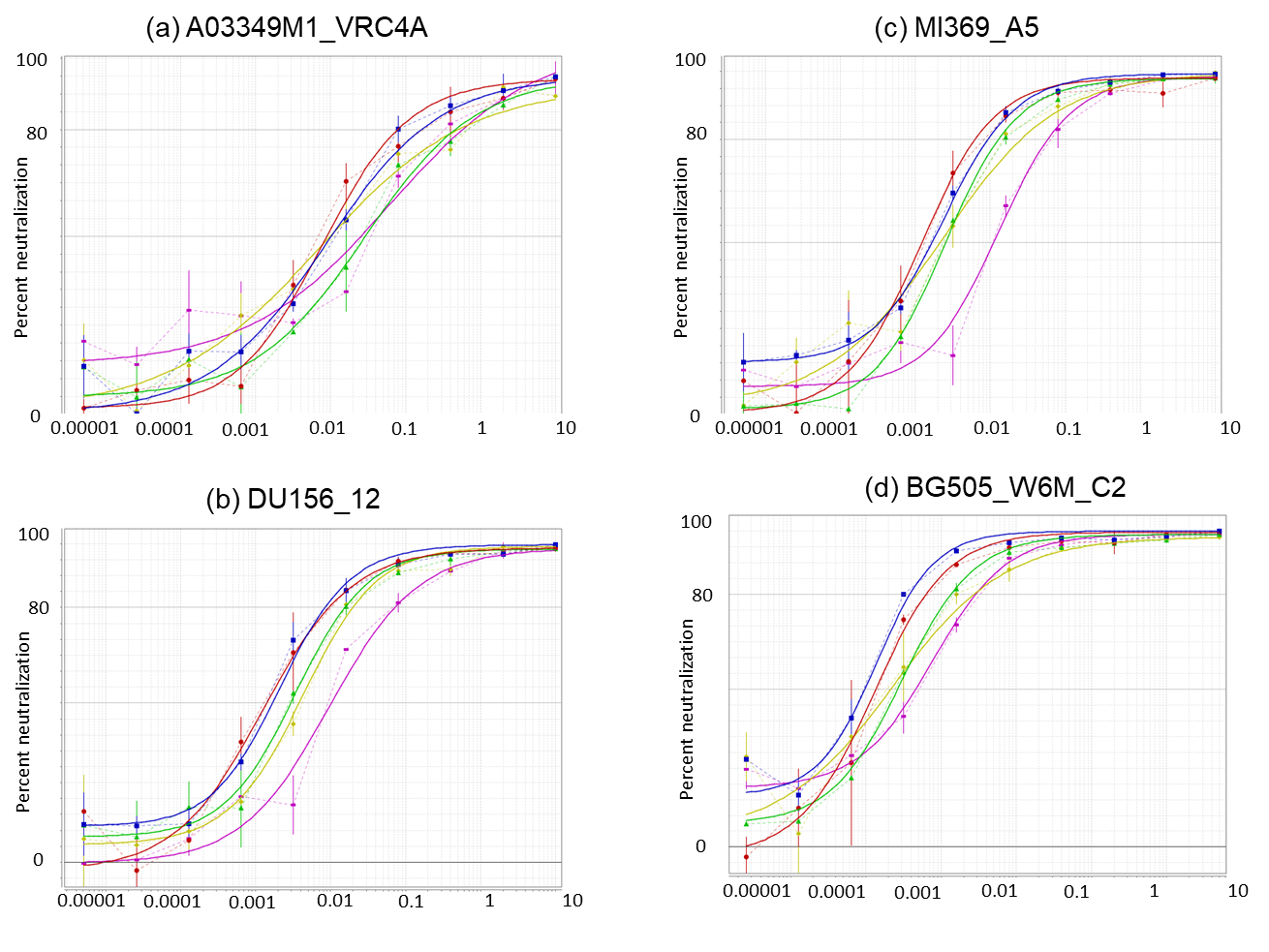


**
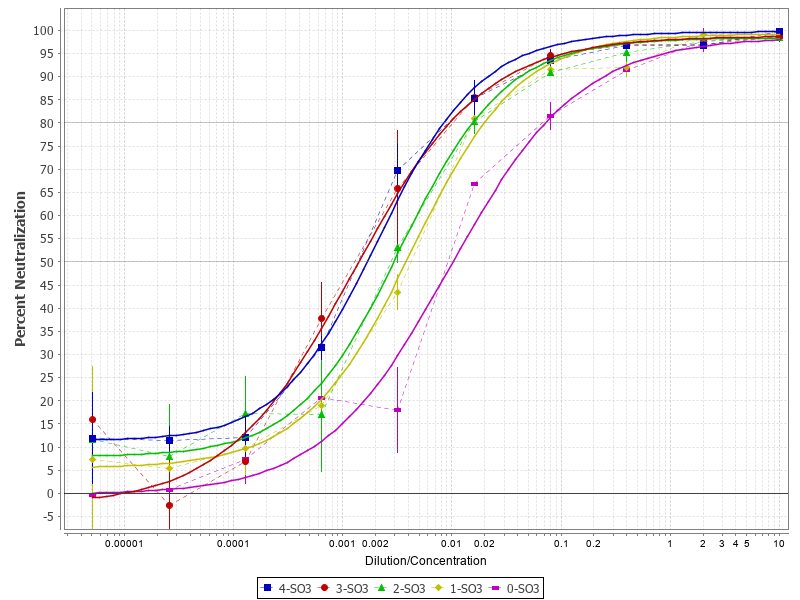
**

Figure S3 Neutralization plots from Labkey analysis of five different levels of sulfation for four panel of virus, (a) A03349M1_VRC4A, (b) Du156_12, (c) MI369_A5, and (d) BG505_W6M_C2.


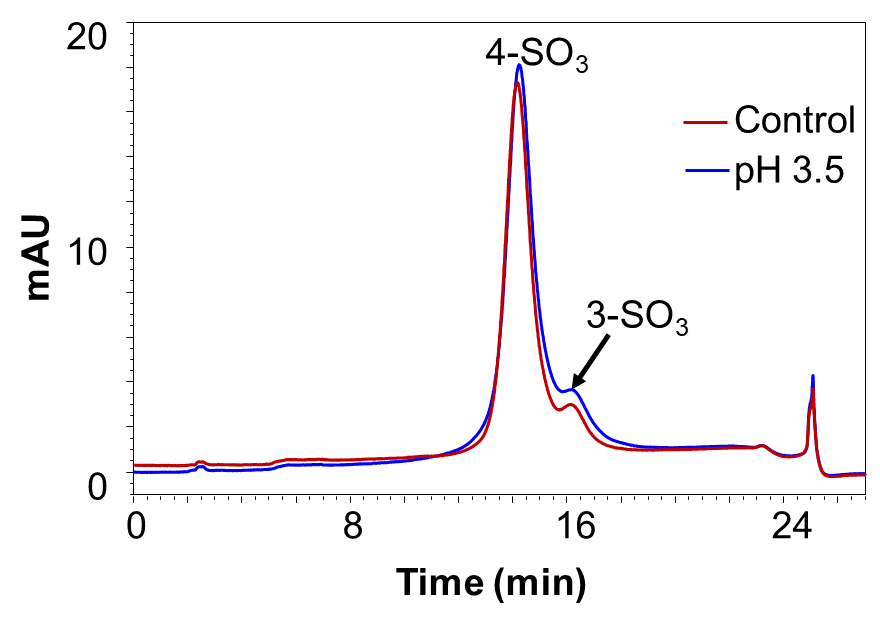


Figure S4 CAP256V2LS sulfation is not altered by low pH. UV-overlays of CAP256V2LS control (before incubation) and pH stressed sample (pH 3.5, incubated at ambient temperature for 2 hrs). No significant changes of main peak 4-SO_3_ were observed.

Table S3 HIC-UV Monitoring 4-SO_3_% levels for CAP256V2LS during elevated temperature stress.

| **Sample CAP256V2LS** | **Temperature (°C)** | **Incubation time** | **% 4-SO3** | **Difference to starting CAP256V2LS (%)** |
| --- | --- | --- | --- | --- |
| **Starting material** | -80 | NA | 89.7 | NA |
| **Stressed materials by thermolysis** | 25 | 1 week | 89.6 | 0.1 |
|  |  | 2 weeks | 89.7 | 0.1 |
|  | 40 | 1 week | 89.8 | 0.1 |
|  |  | 2 weeks | 90.8 | 1.1 |

Table S4 Neutralization efficiency against small panel of HIV viruses for 5 CAP256V2LS lots.

| **Lot ID** | **IC_80_(µg/mL)** | | | |
| --- | --- | --- | --- | --- |
|  | **A03349M1_VRC4A** | **DU156.12** | **MI369** | **SVAMLV** |
| **Reference Lot** | 0.0626 | 0.0059 | 0.0047 | >5 |
|  | 0.0572 | 0.0083 | 0.0057 | >5 |
|  | 0.0409 | 0.0065 | 0.0075 | >5 |
|  | 0.0472 | 0.0079 | 0.0072 | >5 |
|  | 0.0617 | 0.0058 | 0.0103 | >5 |
|  | 0.0395 | 0.0079 | 0.0104 | >5 |
| **Tox Lot** | 0.0630 | 0.0092 | 0.0058 | >5 |
|  | 0.0466 | 0.0078 | 0.0071 | >5 |
|  | 0.0953 | 0.0127 | 0.0084 | >5 |
|  | 0.0568 | 0.0152 | 0.0068 | >5 |
|  | 0.1180 | 0.0100 | 0.0105 | >5 |
|  | 0.0612 | 0.0071 | 0.0081 | >5 |
| **Consistency Lot 1** | 0.0490 | 0.0050 | 0.0047 | >5 |
|  | 0.0466 | 0.0055 | 0.0080 | >5 |
|  | 0.0478 | 0.0050 | 0.0049 | >5 |
|  | 0.0754 | 0.0049 | 0.0111 | >5 |
|  | 0.0415 | 0.0083 | 0.0061 | >5 |
|  | 0.0812 | 0.0052 | 0.0082 | >5 |
| **Consistency Lot 2** | 0.0489 | 0.0060 | 0.0054 | >5 |
|  | 0.0623 | 0.0054 | 0.0054 | >5 |
|  | 0.0992 | 0.0089 | 0.0095 | >5 |
|  | 0.0580 | 0.0096 | 0.0094 | >5 |
|  | 0.0706 | 0.0057 | 0.0094 | >5 |
|  | 0.0333 | 0.0057 | 0.0092 | >5 |
| **Consistency Lot 3** | 0.0380 | 0.0073 | 0.0055 | >5 |
|  | 0.0396 | 0.0067 | 0.0059 | >5 |
|  | 0.0819 | 0.0068 | 0.0074 | >5 |
|  | 0.0561 | 0.0086 | 0.0077 | >5 |
|  | 0.0431 | 0.0067 | 0.0069 | >5 |
|  | 0.0635 | 0.0050 | 0.0062 | >5 |


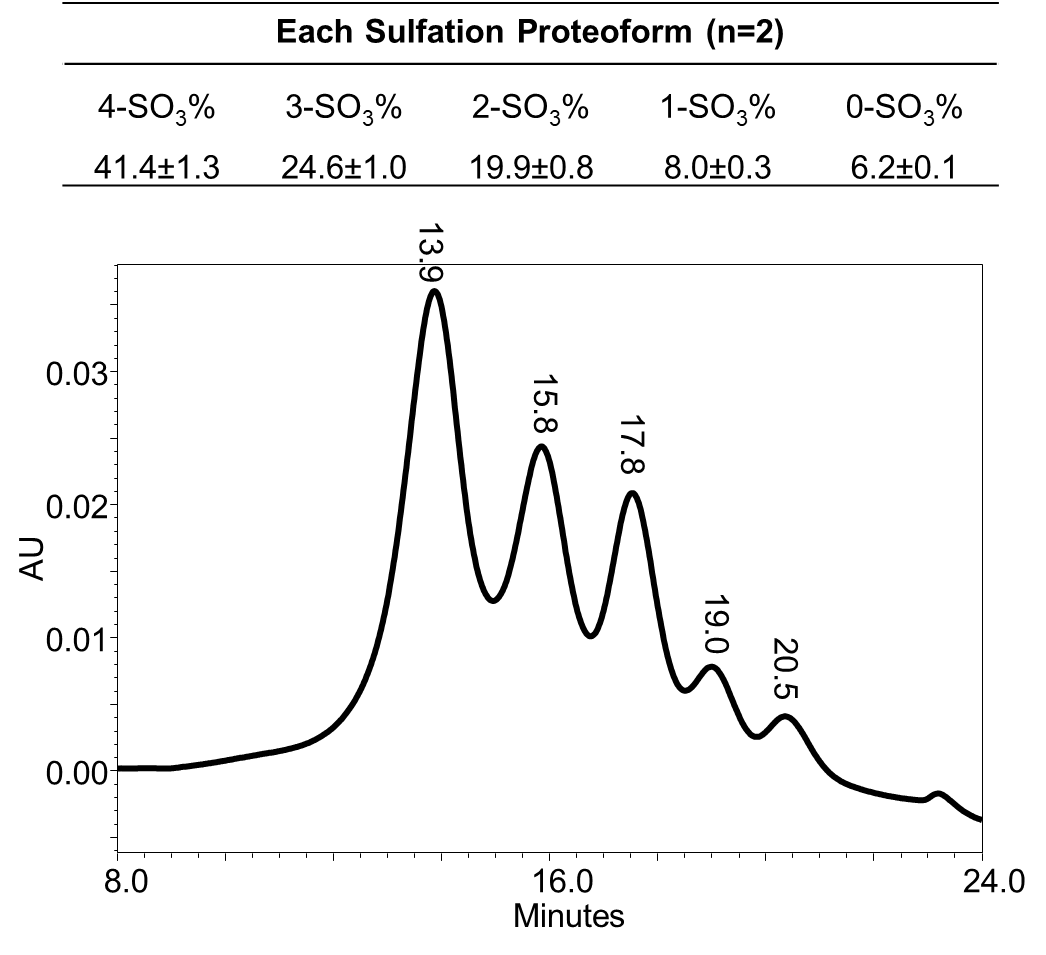


Figure S5 CAP256V2LS sulfation profile was detected for CAP256V2LS HEK-293 material using HIC-UV method. 4-, 3-, 2-, 1- and 0-SO_3_ eluted at 13.9 min (41.4%), 15.8 min (24.6%), 17.8 min (19.9%), 19.0 min (8.0%), and 20.5 min (6.2%), respectively.

Subunit analysis: IdeS proteolysis followed by a reduction was used to characterize the smaller fragments of CAP256V2LS using RPLC-MS, which led to a sulfation profiling of each Fd arm.

Method: 20 µg of each deglycosylated CAP256V2LS sample was first incubated with 50 units of IdeS (Promega, Madison, WI) at 37 ^°^C (pH 7.8) for 30 min and then followed by a reduction with 25 mM DTT at 37 ^°^C for 30 min. Samples were injected onto a BEH C8 column (Acquity, 1.7 µm, 2.1 mm x 50 mm) heated at 80 ^°^C. The same mobile phases for intact mass analysis were applied and delivered at 0.2 mL/min. The RPLC gradient was set as following (time-B%): 0 min-25%, 2 min-25%, 23 min-36.9%, 23.1 min-90%, 25 min-90%, 25.1 min-25%, and 28 min-25%.


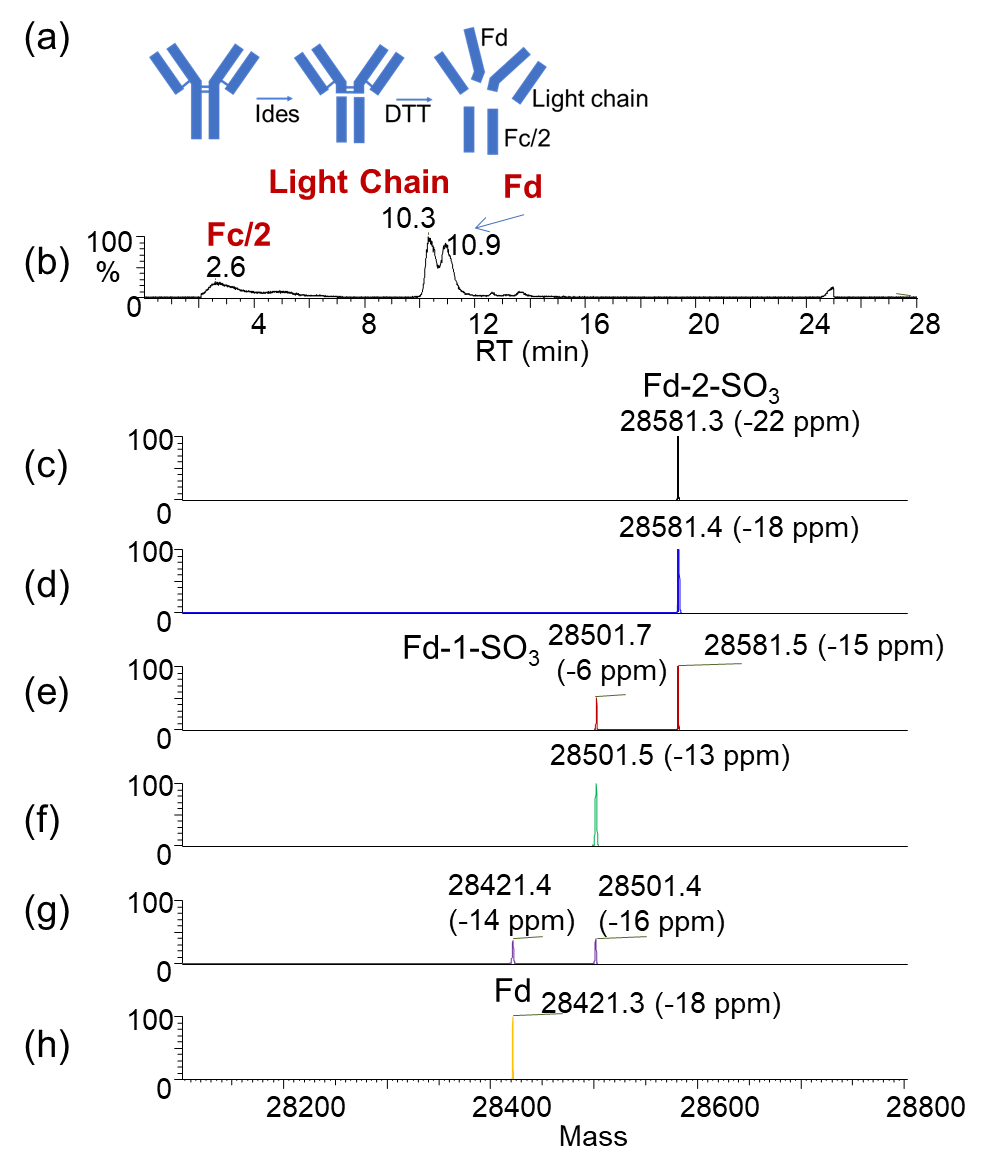


Figure S6 Analysis of IdeS proteolysis Fd sulfation profile for different fractions. Deglycosylated CAP256V2LS (clone 386) was (a) digested using IdeS and reduced by DTT to generate Fc/2, light chain and Fd. During RPLC-MS analysis, (b) a representative TIC showed that Fc, light chain and Fd eluted at 2.6 min, 10.3 min and 10.9 min, respectively. (c) Unfractionated CAP256V2LS showed the main peak at 28581.3 Da correlated to Fd with 2-SO_3_ (Fd-2-SO_3_), but neither Fd-1-SO_3_ nor Fd-0-SO_3_ was detected. It was potentially due to the low intensity of those fragments is below current method detection limit. (d) For the fraction with 4-SO_3_, only Fd arm containing two sulfation (Fd-2-SO_3_) was detected as expected. (e) In 3-SO_3_ fraction, Fd-1-SO_3_ and Fd-2-SO_3_ show the similar peak intensity as predicted. (f) In 2-SO_3_ fraction, Fd-1-SO_3_ was the main peak. Neither Fd-2-SO_3_ nor Fd-0-SO_3_ peak was observed, which indicates the presence of the main structure with sulfation equally distributed at each Fd arm. (g) 1-SO_3_ fraction is expected to have 1 and 0 sulfation at each Fd, which matched the testing results. (h) Only Fd without sulfation should be detected in 0-SO_3_ fraction, which was observed.
